# Ultrasound Imaging in Knee Osteoarthritis: Current Role, Recent Advancements, and Future Perspectives

**DOI:** 10.3390/jcm13164930

**Published:** 2024-08-21

**Authors:** Valerio D’Agostino, Angela Sorriento, Andrea Cafarelli, Danilo Donati, Nicolas Papalexis, Alessandro Russo, Gina Lisignoli, Leonardo Ricotti, Paolo Spinnato

**Affiliations:** 1 Diagnostic and Interventional Radiology, IRCCS Istituto Ortopedico Rizzoli, Via GC Pupilli 1, 40136 Bologna, Italy; 2 Radiology Unit, Policlinico Ospedaliero “Umberto I”, Nocera Inferiore, 84014 Salerno, Italy; 3 The BioRobotics Institute, Scuola Superiore Sant’Anna, 56127 Pisa, Italy; 4 Department of Excellence in Robotics & AI, Scuola Superiore Sant’Anna, 56127 Pisa, Italy; 5 Clinical and Experimental Medicine PhD Program, University of Modena and Reggio Emilia, 41121 Modena, Italy; 6 Clinica 2, IRCCS Istituto Ortopedico Rizzoli, 40136 Bologna, Italy; 7 Laboratorio di Immunoreumatologia e Rigenerazione Tissutale, IRCCS Istituto Ortopedico Rizzoli, 40136 Bologna, Italy

**Keywords:** knee, osteoarthritis, ultrasound imaging, ultrasonography, interventional, telemedicine, narrative review

## Abstract

While conventional radiography and MRI have a well-established role in the assessment of patients with knee osteoarthritis, ultrasound is considered a complementary and additional tool. Moreover, the actual usefulness of ultrasound is still a matter of debate in knee osteoarthritis assessment. Despite that, ultrasound offers several advantages and interesting aspects for both current clinical practice and future perspectives. Ultrasound is potentially a helpful tool in the detection of anomalies such as cartilage degradation, osteophytes, and synovitis in cases of knee osteoarthritis. Furthermore, local diagnostic and minimally invasive therapeutic operations pertaining to knee osteoarthritis can be safely guided by real-time ultrasound imaging. We are constantly observing a growing knowledge and awareness among radiologists and other physicians, concerning ultrasound imaging. Ultrasound studies can be extremely useful to track the response to various therapies. For this specific aim, tele-ultrasonography may constitute an easy tool aiding precise and repeated follow-up controls. Moreover, raw radio-frequency data from US backscattering signals contain more information than B-mode imaging. This paves the way for quantitative in-depth analyses of cartilage, bone, and other articular structures. Overall, ultrasound technologies and their rapid evolution have the potential to make a difference at both the research and clinical levels. This narrative review article describes the potential of such technologies and their possible future implications.

## 1. Introduction

### 1.1. Osteoarthritis

Osteoarthritis (OA) represents the predominant joint pathology in the adult population worldwide [1]. Research by Felson et al. indicates that approximately one-third of adults exhibit radiographic evidence of OA, contrasting with the findings of Andrianakos et al., who identified clinically significant OA in only 8.9% of the adult populace through epidemiological investigation [2,3]. This casts a shadow on the clinical relevance of the radiologic signs of OA.

Furthermore, OA stands as a primary cause of pain and functional impairment among adults [4,5], with 80% of afflicted individuals encountering mobility restrictions and 25% experiencing substantial limitations in their daily activities [6].

Although OA is ubiquitous, its etiology and pathophysiology are poorly understood. Nowadays, OA is seen as the clinical and pathologic outcome of a range of disorders that result in structural and functional failure of synovial joints. Inflammatory changes, unlike those observed in rheumatoid or psoriatic arthritis, are likely secondary due to the soluble breakdown of cartilage and bone. Researchers increasingly view OA not as a passive degenerative condition but as an active process primarily influenced by biomechanical factors [7]. Other factors, such as genetic predisposition, metabolic abnormalities, and possibly vascular abnormalities, also seem to contribute to its development, especially in the early stages of the disease [8,9].

Its clinical presentation is heterogeneous both in grade and timing, with typical symptoms including pain, stiffness, and movement restriction.

OA is a complex chronic disorder, with therapeutic strategies primarily focusing on palliative and reactive approaches [10]. Non-pharmacological interventions, including education, self-management, exercise, weight management for those overweight or obese, and appropriate use of assistive devices, constitute first-line treatment recommendations [11]. Recently, some clinical trials have shown promising results with drugs that can positively modify the structural progression of the disease, the so-called disease-modifying osteoarthritis drugs (DMOADs) [12,13].

In severe cases, surgical interventions such as joint replacement or osteotomy may be necessary [14,15].

### 1.2. Knee Osteoarthritis

Among all the joints affected by OA, the knee is the most common location (6% of all adults) [3]. As a matter of fact, the likelihood of developing OA increases with age. Research findings indicate a notable prevalence asymmetry of knee OA among men aged 60 to 64, with a higher occurrence in the right knee (23%) compared to the left knee (16.3%). Conversely, in women of the same age group, the distribution appears more evenly balanced, with rates of 24.2% in the right knee and 24.7% in the left knee [3,16].

The prevalence of knee OA escalates notably among individuals aged 70 to 74, reaching up to 40% [2]. However, when relying solely on clinical criteria for diagnosis, the prevalence diminishes to 10% among adults [3]. Estimates from 2012 indicate that approximately 250 million individuals globally were afflicted with knee OA [17]. Considering the combined impact of aging and escalating obesity rates, projections suggest that the proportion of individuals aged 45 years or older diagnosed with knee OA by a physician could surge from 13.8% to 15.7% [18].

Although cartilage degeneration and osteophyte formation remain the structural hallmarks of knee OA, the disease is now increasingly recognized as a whole-organ disorder affecting tissues in the entire knee joint, such as the meniscus and synovium [19]. This has led to the necessity of other imaging methods to better comprehend the disease burden.

### 1.3. Imaging in Knee Osteoarthritis (OA)

Although imaging is used to support a clinical diagnosis of OA [20], advancements in imaging technologies have significantly enhanced our comprehension of OA by providing insight into the various structures in the joint involved, enabling and facilitating the evaluation of structural changes and establishing links with clinical manifestations [21].

The Osteoarthritis Research Society International (OARSI)–FDA initiative further classified OA-specific biomarkers into two major groups: wet biomarkers and dry biomarkers. Wet or soluble biomarkers represent a modulation of endogenous substances in body fluids and are measured in urine, blood, serum, plasma, or synovial fluid. Dry biomarkers include imaging markers of radiography, ultrasonography, magnetic resonance imaging (MRI), and others [22].

Although conventional radiography (CR) remains the traditional modality of imaging for assessing OA, it suffers from notable limitations such as low sensitivity and limited clinical correlation. Emerging modalities like magnetic resonance imaging (MRI) and ultrasound offer promise in overcoming these shortcomings, providing more precise visualization of bone and soft tissue abnormalities that are pivotal in OA research. However, the exact role of imaging in both clinical practice and research in the assessment of knee OA remains ambiguous, and the widespread availability of these modalities has raised concerns regarding potential overutilization, resulting in excessive healthcare burdens and costs [23].

The present narrative review aims to summarize existing evidence on the use of different imaging modalities in evaluating knee OA, with a focus on novel applications of ultrasound and future perspectives on the potential of this technique.

#### 1.3.1. Conventional Radiography

OA structural changes have traditionally been assessed with CR [24]. Today, CR is still the modality of choice to confirm a structural diagnosis of OA and to monitor its progression. However, the radiological demonstration of typical signs of OA of the knee is poorly correlated with symptoms; only about 15% of patients with radiologically demonstrated knee OA complain of knee pain [16].

The standard approach consists of weight-bearing posteroanterior knee radiographs obtained in a fixed flexion position of the symptomatic knee with 20°–30° flexion and 10° external rotation of the feet positioning.

Radiographs serve as a valuable tool for visualizing the bony characteristics of OA, including marginal osteophytes, subchondral sclerosis, and subchondral cysts. Furthermore, they indirectly assess cartilage thickness and meniscal integrity by observing Tibiofemoral joint space width (JSW). Disease severity evaluation via radiography relies on osteophyte presence and joint space narrowing (JSN). Osteophytes typically precede the development of JSN. However, radiography presents limitations such as challenges in reproducing consistent positioning across visits, moderate sensitivity for detecting temporal changes, and poor specificity in assessing soft tissue damage contributing to OA-related pain. To gauge radiographic OA severity, semiquantitative scoring systems are employed, often referencing published atlases depicting various OA grades [25,26,27]. The Kellgren and Lawrence (K–L) grading system, established in 1957, is the standard for diagnosing radiographic OA, basing diagnoses on the presence of a “definite” marginal osteophyte on weight-bearing radiographs [24]. However, the K–L system has limitations, including assumptions about sequential disease progression, composite measures of JSW and osteophytes, and a lack of differentiation between medial and lateral tibiofemoral disease. In contrast, the OARSI atlas classification separately scores JSN and osteophytes in each knee compartment [25,26]. Standardized knee positioning techniques, aiming for consistent knee flexion, are crucial for reproducible measurements [28,29]. While manual JSW measurement methods are straightforward, automated, and semiautomated techniques offer rapid, objective, and precise assessments [30,31,32]. Despite advancements in 3D imaging, radiographically assessed JSW loss remains the recommended structural outcome for demonstrating the efficacy of disease-modifying OA drugs (DMOADs) in phase III clinical trials, per regulatory agencies like the FDA. Nonetheless, it is important to mention that radiographic JSW measurements do not exclusively reflect cartilage damage [33].

#### 1.3.2. Magnetic Resonance Imaging

**Figure 1:**
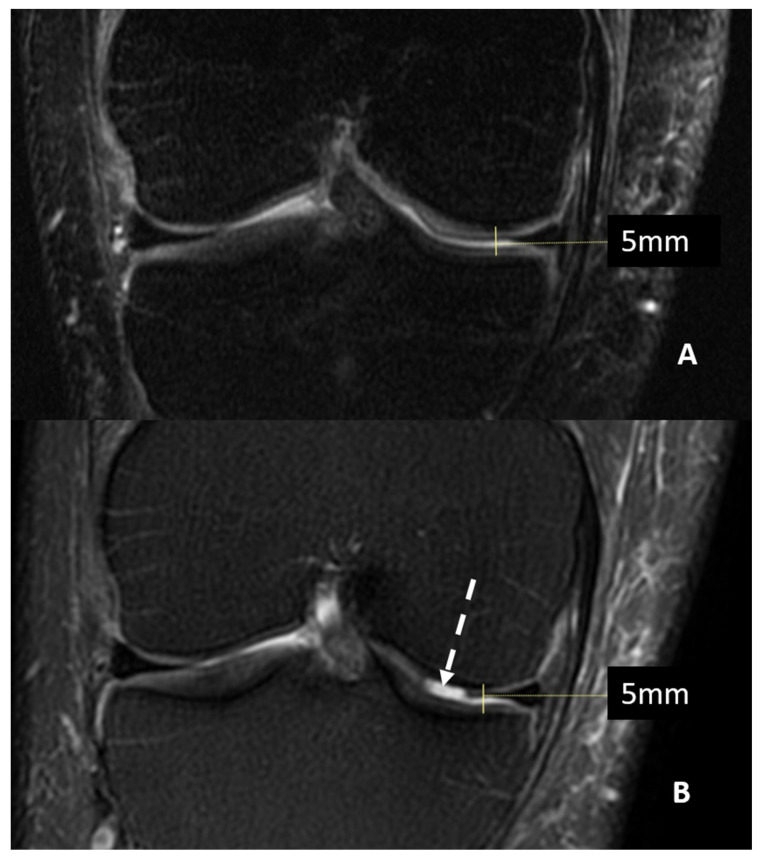
MRI (T2w fat saturated coronal sequences) of a patient with suspected knee osteoarthritis at baseline (Panel **A**) and seven years later (Panel **B**). A progression of cartilage damage over time can be noted, especially at the medial femur condyle, where a large defect is detectable (arrow, Panel **B**), even if joint space thickness remains the same.

Magnetic Resonance Imaging (MRI) can offer valuable insight into inner structures and cartilage surfaces of the knee joint, with assessments that are both semi-quantitative and qualitative (Figure 1).

The quantitative assessment of cartilage morphology relies on the three-dimensional (3D) nature of MRI data, enabling the analysis of morphological tissue parameters as continuous variables [34,35]. Specialized image analysis software is employed to calculate various morphological parameters across multiple subregions, encompassing cartilage volume, thickness, subchondral bone size, cartilage surface area, and the delineation of regions of denuded and cartilage-covered subchondral bone, among others [36]. Accurate quantitative measurement of cartilage morphology necessitates standardized high-resolution gradient-echo 3D imaging sequences, such as T1-weighted spoiled gradient-echo (SPGR) or double-echo steady-state (DESS) images with fat suppression or water excitation, which offer superior contrast for delineating the bone-cartilage interface and cartilage surface and exhibit heightened sensitivity to longitudinal changes [37]. Quantitative cartilage morphometry has found application across diverse sample populations, including participants from the Osteoarthritis Initiative (OAI) with up to four-year follow-up data [37,38], and in clinical Disease-Modifying Osteoarthritis Drug (DMOAD) trials with observation periods of up to five years [39]. Traditionally, cartilage morphometry serves to gauge longitudinal changes in knees afflicted with established radiographic OA, as they are more prone to manifest structural progression [33]. Consequently, patients are often selected based on the severity of radiographic disease, as defined by scoring systems such as the Kellgren–Lawrence (K–L) system.

Noteworthily, early-stage OA typically lacks substantial cartilage loss, although minor changes may be discerned using location-independent analysis methods [40]. Early OA cohorts may exhibit localized thinning and thickening at distinct locations, complicating the analysis of changes in cartilage morphology at the whole joint or plate level [41]. To surmount these challenges, location-independent analyses focusing on the magnitude of change in cartilage thickness, irrespective of location, have been proposed. These approaches, such as the “ordered values” approach or thinning/thickening scores, disentangle the magnitude and location of change, thereby enhancing sensitivity to differences in change [42]. They have demonstrated enhanced discrimination between healthy and OA subjects, superior performance in detecting risk factors for OA progression, and heightened sensitivity to treatment interventions [43,44,45].

Expert semiquantitative MRI scoring constitutes a valuable approach for evaluating knee OA in both observational studies and clinical trials. The Whole-Organ Magnetic Resonance Imaging Score (WORMS) [46] spearheaded a comprehensive whole-organ approach to knee OA assessment, covering a wide spectrum of tissues implicated in the OA disease process. Over the ensuing two decades, several additional whole-organ knee scoring systems, including the Knee Osteoarthritis Scoring System (KOSS) [47], Boston Leeds Osteoarthritis Knee Score (BLOKS) [48], and MRI Osteoarthritis Knee Score (MOAKS) [49], have been developed. Intrareader and inter-reader reliability assessments for semiquantitative measures have consistently demonstrated moderate to excellent reliability across various studies, bolstering the credibility of semiquantitative MRI scoring as a robust assessment tool [50]. This approach, coupled with rapid MRI assessment and accelerated image acquisition techniques, holds promise for utilizing MRI as a routine screening tool for stratifying patients based on their structural characteristics in OA clinical trials [51].

#### 1.3.3. Computed Tomography

Computed tomography (CT) imaging, compared to MRI, offers superior spatial resolution and excellent multiplanar capabilities, although its ability to provide tissue contrast is somewhat limited. It is particularly effective in identifying specific structural features of joint degeneration in OA patients, including osteophytes, subchondral cysts, bone sclerosis, joint effusion, and periarticular cysts [52]. CT demonstrates heightened sensitivity in detecting osteophytes, presenting them as larger in size compared to standard fat-suppressed FSE MRI scans [53]. CT arthrography, which utilizes intra-articular iodinated contrast, remains a valuable tool for measuring cartilage thickness and accurately identifying cartilage defects, meniscus tears, and anterior cruciate ligament (ACL) injuries within the knee joint. It also enables simultaneous assessment of cartilage thickness and subchondral bone mineral density, allowing for the exploration of regional interactions between cartilage and bone in OA development and progression [54,55]. Furthermore, CT arthrography provides insights into cartilage proteoglycan content by tracking the diffusion of anionic intra-articular iodinated contrast, which is inversely proportional to the concentration of negatively charged glycosaminoglycans in the macromolecular matrix [56]. However, CT arthrography is an invasive procedure and may not effectively detect certain structural features of joint degeneration, such as joint effusion, synovitis, and bone marrow lesions (BMLs). Consequently, its widespread adoption in OA has been limited. Recent technological advancements have introduced novel CT applications for structural OA assessment. Dual-energy CT (DECT) can differentiate structures with similar densities but distinct elemental compositions, enhancing the characterization of crystal deposition diseases implicated in knee OA progression [57,58]. Additionally, DECT can generate bone subtraction images to identify attenuation changes within bone marrow associated with posttraumatic and degenerative bone marrow edema lesions [59]. Extremity cone beam CT represents another innovative development, offering high-resolution weight-bearing imaging of the knee and ankle with minimized radiation exposure [60,61]. Weight-bearing CT allows for a comprehensive assessment of structural aspects of knee joint degeneration and exhibits high scan-rescan reliability in measuring tibiofemoral joint space width (JSW) [62]. These measurements correlate strongly with those obtained through fixed flexion radiography and hold clinical significance in influencing symptoms and physical function in knee OA subjects [63].

## 2. Ultrasound Imaging for Knee Osteoarthritis Assessment

### 2.1. Current Clinical Role

**Figure 2:**
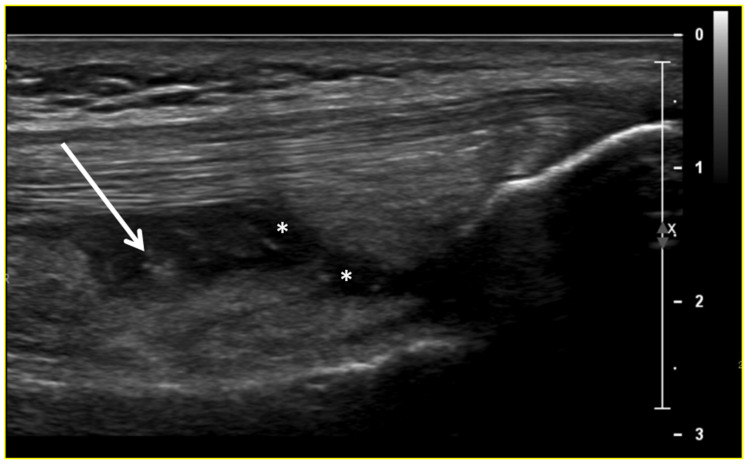
Ultrasound image (B-mode) longitudinal suprapatellar view showing joint effusion (asterisks) and synovial inflammatory thickening (arrow) within the sub-quadricipital recess.

**Figure 3:**
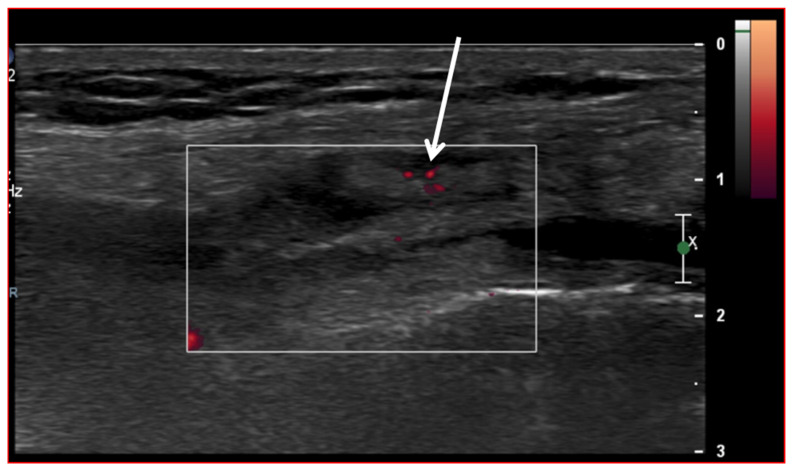
Ultrasound image (Power-Doppler) longitudinal, lateral suprapatellar view showing synovial inflammatory foci with inflammatory hyperemia (arrow).

**Figure 4:**
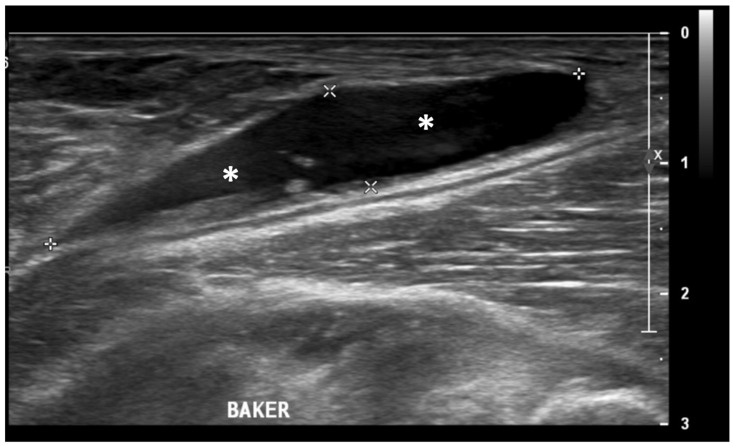
Ultrasound image (B-mode) longitudinal posterior view in the medial aspect of the popliteal fossa showing large fluid collection (asterisks) within the medial head of the gastrocnemius and the semimembranosus tendons’ sheet (Baker’s cyst).

Ultrasound (US) imaging has not traditionally been used as part of the clinical pipeline for OA diagnostics. However, when examining the scientific literature, its role has risen steadily during the last two decades. US assessment of joints offers several advantages, including the ability to assess soft-tissue changes associated with OA and to outline the contour of the bony surface surrounding the joint [64,65]. In addition to detecting structural OA changes, a US can provide insights into inflammatory findings, complementing traditional CR imaging [66]. Several inflammatory findings (e.g., joint effusion/Baker cyst, synovial thickening, and hyper-vascularity), easily detectable throughout a US, are associated with pain exacerbation and disease progression in knee OA (Figure 2, Figure 3 and Figure 4).

However, a major limitation of US joint assessment is the inability to visualize intra-articular structures. Furthermore, US imaging is cost-effective and widely available in many primary healthcare facilities worldwide. As a result, a US can be considered a complementary tool alongside CR [67]. Thus, a US presents an intriguing approach to modern OA imaging and may serve as a valuable addition to the clinical toolkit for OA diagnostics [68].

### 2.2. Ultrasound and Knee Cartilage Assessment

**Figure 5:**
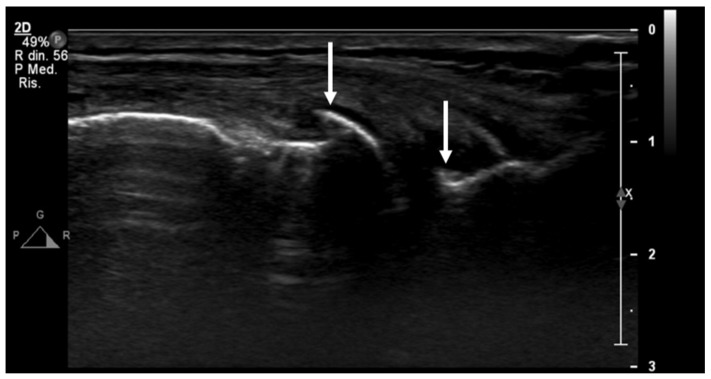
An ultrasound image (B-mode) longitudinal view of the medial aspect of the knee shows initial signs of osteoarthritis, with small osteophytes of the femur and tibiae epiphyses (arrows).

The majority of research on US assessment of OA primarily focuses on the knee joint. Generally, US demonstrates reliable capability in evaluating tibiofemoral osteophytes (Figure 5), effusion/synovitis, and meniscus protrusion, particularly in the medial compartment.

**Figure 6:**
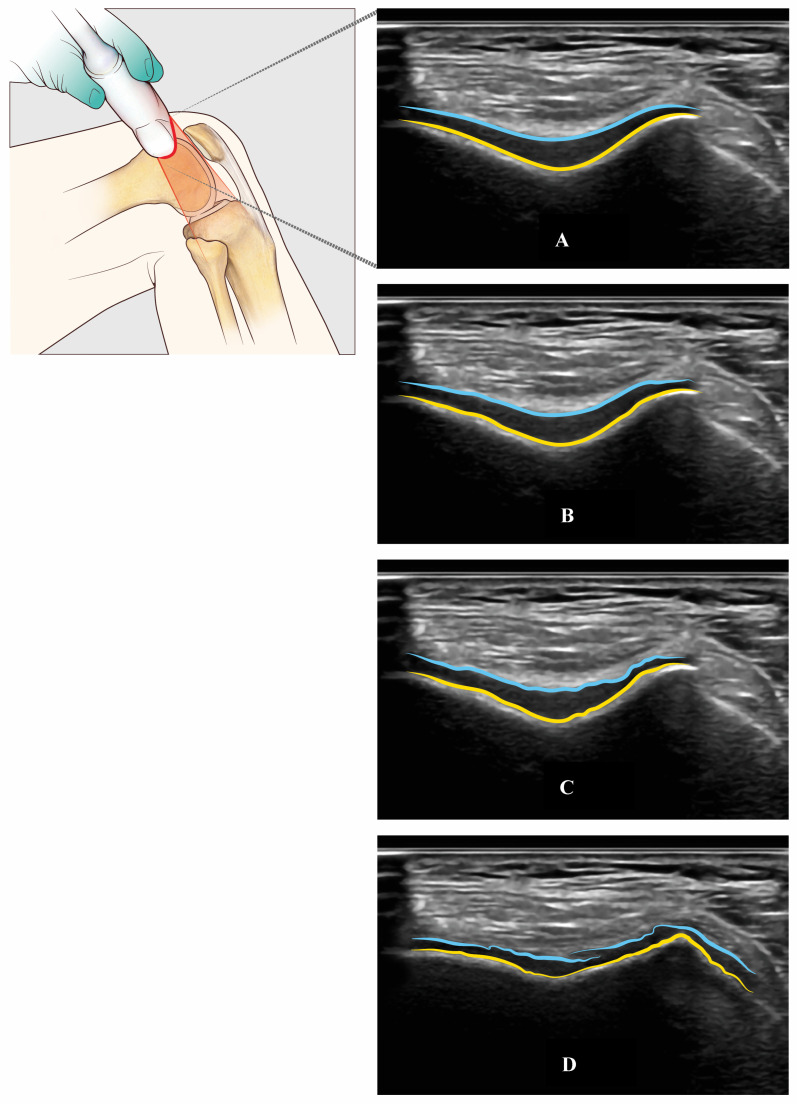
Trochlear femur’s cartilage assessed with US through an axial suprapatellar view with a flexion of the knee (light blue, superficial layer of the cartilage—yellow, cortical bone of the trochlear groove): Normal cartilage (Panel **A**), mild (Panel **B**), moderate (Panel **C**), and severe (Panel **D**) cartilage damage.

A US’s capability of assessing articular cartilage is somewhat limited and not yet fully explored. Specifically, a US provides only a restricted view of the femoral condylar cartilage, depending on patellar position, size, and morphology. On the contrary, a US is able to view all the trochlear femur cartilage through a suprapatellar view with the knee in maximal flexion (Figure 6).

To address this limitation, Kauppinen et al. [69] conducted a study involving 20 healthy knees, utilizing both US and 0.6 mm isotropic MRI with a 90-degree flexed knee to evaluate the ability of US to visualize femoral articular cartilage. Their findings suggested that up to two-thirds of the articular cartilage of the medial femoral condyle and one-third of the lateral femoral condyle could be assessed by US. Several studies have compared US findings with surgical gold standards. For instance, Saarakkala et al. [70] investigated 40 patients using knee arthroscopy as the gold standard and found significant associations of cartilage changes between US and arthroscopy, particularly at the sulcus and medial femoral condyle. Subsequently, Nevalainen et al. [71] utilized total knee arthroplasty as a gold standard in a series of 57 late-stage knee OA patients, demonstrating excellent sensitivity of US, especially on the medial aspect of the knee joint.

Histological studies, such as the work conducted by Lee et al. [72], have also shown significant correlations between in vivo US grading of cartilage and histological grading.

Furthermore, several studies have explored the prognostic value of US findings in knee OA. Ishibashi et al. [73] followed 404 subjects for three years and found associations between OA progression, female sex, body mass index, and knee effusion. Chiba et al. [74] conducted a 5-year follow-up cohort study of 944 knees, revealing that greater medial meniscus extrusion predicted a higher prevalence of radiographic OA. Sarmanova et al. [75] challenged existing cut-offs for effusion and synovial hypertrophy, suggesting broader normal ranges. Additionally, Kawaguchi et al. [76,77] observed an association between medial meniscus extrusion, weight-bearing, and higher Kellgren-Lawrence grade. Bevers et al. and Conaghan et al. [78] reported associations between US-detected effusion and synovial hypertrophy with radiological and clinical progression, as well as knee arthroplasty, respectively.

The correlation between US findings and clinical symptoms has been a major topic of research. Abicalaf et al. studied 194 OA knees showing a significant moderate positive association between VAS scores, WOMAC scores, and the number of US findings [79]. Philpott et al. studied the association between US-detected synovitis (both grayscale and Power Doppler) and pain in 248 knee OA patients (a total of 453 knees), concluding that moderate or severe synovitis was strongly associated with constant pain [80].

Furthermore, the reliability of a US in assessing knee OA has consistently been found to be good to excellent over the years. Both intra- and inter-rater agreements have been extensively evaluated in multiple studies. In a recent study involving 89 subjects, Oo et al. demonstrated excellent inter-rater agreement in quantitative US evaluations for osteophytes (ICC range = 0.90–0.96), meniscal extrusion (ICC range = 0.90–0.93), and synovitis (ICC range = 0.86–0.88) [81]; Razek et al. obtained similar results, with an excellent inter-rater agreement (k = 0.86–1.00) with 80 knee OA patients [82].

### 2.3. Tele-Ultrasonography

US is the imaging technology most suitable for telemedicine due to its non-invasive nature, real-time imaging capabilities, and portability. Tele-ultrasonography (TUS) involves performing and interpreting US examinations remotely using telecommunication technologies. Synchronous TUS features real-time communication between the US operator and the remote expert radiologist, enabling immediate interpretation and feedback on US images as they are acquired. In contrast, asynchronous TUS involves capturing US images and data, which are then transmitted and stored for later remote interpretation by the expert [83].

TUS allows for remote diagnostics and monitoring, enabling healthcare providers to assess and manage patient conditions without requiring in-person visits. In recent years, advancements in TUS have been recognized as an effective solution to address the limited access to specialized US consultations, especially in high-income settings and low-resource areas [84].

TUS has already been employed to provide remote training and education [85]. A novice ultrasound operator can be effectively guided by a remote expert radiologist through specific ultrasound protocols, allowing the radiologist to evaluate the diagnostic quality of the acquired data remotely. Numerous promising results have demonstrated the potential of TUS to significantly enhance care and treatment across a range of clinical fields, including emergency medicine, obstetrics and gynecology, hemophilia management, cardiology, pediatric cardiac imaging, US-guided nerve blocks, human immunodeficiency virus-associated tuberculosis and cystic echinococcosis and musculoskeletal injuries [86]. In the musculoskeletal domain, TUS has been explored to monitor various anatomical regions, such as elbow, forearm, foreleg, knee, and ankle, and to evaluate shoulder integrity in space [87,88].

**Figure 7:**
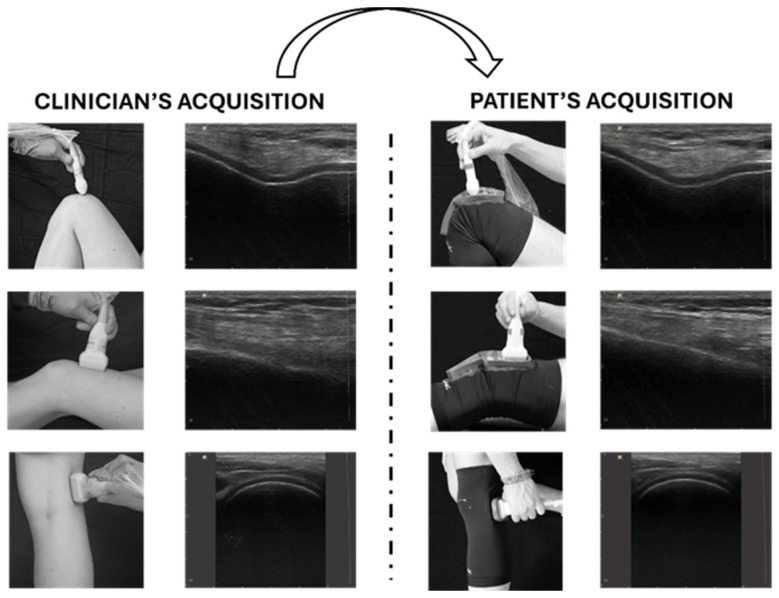
Tele-ultrasonography approach. The figure presents a schematic representation of the asynchronous tele-ultrasonography method proposed in recent research [89]. The clinician (**left** panel) acquires reference standard images for knee evaluation; the subject (**right** panel) reproduces offline the images of the clinician using specific guidance systems (wearable probe positioner and graphical user interface). Modified from [89].

In the context of OA, a recently published article defined the clinical indications and the standard probe positions for a comprehensive US assessment of knee OA [89]. In this preliminary study, a physical system (namely, a knee brace with specific openings for guiding the correct probe position) was designed, developed, and tested on three inexperienced subjects. This proposed method, depicted in Figure 7, has the potential to facilitate the use of TUS for knee OA, enabling repeatable, non-invasive, cost-effective, and asynchronous assessment of the knee joint. The asynchronous approach offers greater scheduling flexibility, optimizing the clinician’s workload while still providing valuable diagnostic information, albeit without immediate feedback during the examination (Figure 7).

### 2.4. Quantitative Ultrasound

Conventional B-mode US images contain less information than raw radiofrequency (RF) data from US backscattering signals due to extensive filtering processing steps involved in image formation. Quantitative ultrasound (QUS) techniques leverage RF data directly from the piezoelectric elements of a US probe, providing objective and quantitative measures closely related to tissue microstructure and characterization. QUS analysis has been preliminary explored to enhance medical diagnosis of several tissues, including cancer detection, liver disease monitoring, lymph node classification, therapy monitoring, cell death assessment, and bone composition in fracture healing [90,91]. Additionally, QUS has been investigated and clinically validated for assessing bone mineral density in patients with osteoporosis and rheumatoid arthritis [92,93].

In the context of OA, high-frequency ultrasound (20 MHz ≤ f ≥ 50 MHz) has been mostly used to detect the state of cartilage degeneration in various studies. Extracted parameters include time-domain metrics (e.g., speed of sound (SoS), reflection index (RI), surface roughness (URI), and thickness) and frequency-domain parameters (e.g., attenuation coefficient, integrated backscatter coefficient, and integrated reflection coefficient) [94]. Prior studies have shown a strong correlation between backscattered US signals and changes in cartilage content and structure, as reviewed by Nieminen et al. [95]. They reported a change in SoS, attenuation, and backscatter due to the degree of tissue degeneration. SoS decreased in the degenerate cartilage (1570 m/s) with respect to healthy cartilage (1670 m/s). The US attenuation showed a negative correlation with spontaneous cartilage degeneration, while RI was highly dependent on the collagen content and architecture. Saarakkala et al. investigated high-frequency QUS at 20 MHz to analyze ex-vivo bovine articular cartilage subjected to mechanical and enzymatic degradation [96]. They observed that enzymatic treatments induced variations in the acoustic properties of cartilage, such as the RI, URI, and spatial variations in ultrasound reflection. Wang et al. utilized high-frequency ultrasound (40 MHz central frequency) to assess surface integrity, thickness, and acoustic properties of normal and enzymatically degraded articular cartilage [97]. Interesting findings were also observed in OA-induced animal models. Niu et al. demonstrated a strong correlation between acoustic parameters and OA grade in rabbit knees using high-frequency ultrasound (55 MHz central frequency) [98]. However, all these studies employed transducers operating at high frequencies (≥20 MHz), which have limited in-vivo penetration capabilities and can only be clinically applied through integration into arthroscopic probes. Few studies employed lower frequencies (<20 MHz) to detect degenerative changes in cartilage tissue. Zhang et al. examined the effect of the enzymatic degradation induced by trypsin using a frequency of 15 MHz [99]. Three acoustic parameters (IRC, AIB, and averaged magnitude ratio) showed a trend with the loss of proteoglycans due to the trypsin effect. Hattori et al. investigated the enzymatic degradation of collagen content using collagenase, demonstrating an increase in the maximum magnitude of the signal as the degradation increased [100]. Sorriento et al. preliminarily evaluated the effects of two cartilage-degrading enzymes (trypsin and collagenase) by investigating novel parameters associated with the degeneration process and artificial intelligence (AI) algorithms to automatically detect cartilage degeneration [101].

Despite these advancements, QUS techniques are not yet integrated into conventional US machines typically used in clinical settings [90]. This limitation could be mainly attributed to the lack of standardization across the various studies in this field. Hence, there is a pressing need to thoroughly investigate the correlation between QUS parameters and the severity of OA at frequencies typically used for OA diagnosis (approximately 10–15 MHz), aiming at developing non-invasive, safe, quantitative, and reliable diagnostic methods based on a US.

### 2.5. Ultrasound and Therapeutic Opportunities

US is used as a complementary treatment in physical therapy to manage pain and support the healing of soft tissue injuries [102]. This treatment operates through thermal (continuous US) and non-thermal (low-intensity pulsed ultrasound or LIPUS) mechanisms, employing various parameters such as intensity, wavelength, duty cycle, and frequency [103]. Continuous US generates thermal effects, potentially providing pain relief by raising tissue temperature, which increases capillary permeability and tissue metabolism, thus enhancing the extensibility of fibrous tissue and raising pain thresholds. Non-thermal effects, achieved through pulsed US, include modulating cell membrane permeability, boosting protein synthesis, and activating the immune response near the injury site, which can aid in the regeneration of damaged tissue [104,105].

The LIPUS technique has been used for decades for various musculoskeletal conditions (mostly improving fracture repair) [106,107] and was recently proposed as a clinical application in the knee for the stimulation of chondral cells in arthritic subjects [108,109].

The Jo et al. group highlighted an improvement in pain, function, and quality of life in a recent clinical trial, while cartilage thickness measured on MRI did not show statistically significant differences [110].

**Figure 8:**
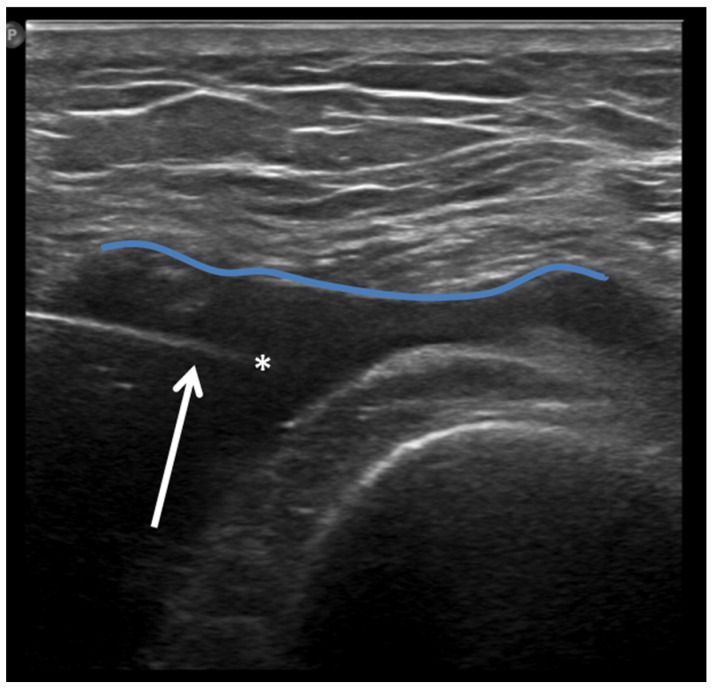
Ultrasound-guided intra-articular lateral needle (20 Gauge—arrow) approach into a suprapatellar recess distention (blue line) with abundant joint fluid collection (asterisk) in a patient with knee osteoarthritis. Fluid aspiration and corticosteroid injection have been performed under ultrasound guidance.

Furthermore, a US is often the first choice for image-guided minimally invasive treatments (e.g., drug injections) in the knee joint due to the wide diffusion and availability of this imaging modality, its low costs, the absence of ionizing radiation, and the possibility of real-time assessment of the needle position (Figure 8).

In the last few years, several studies have highlighted the advantages of imaging guides in joint infiltrations over blind maneuvers (Table 1) [111,112,113,114].

Of course, procedure accuracy depends on the patient operator’s experience and on some of the patient’s body characteristics (grade of knee verism, BMI, deformity, etc.) [115,116,117].

Several trials have analyzed the treatment outcomes with US-guided intraarticular injection in knee OA of different drugs like platelet-rich plasma [118], autologous adipose-derived mesenchymal progenitor cells [119], hyaluronic acid [120], showing similar results in terms of pain reduction and surgery delay when compared [121]. On the other hand, the classical glucocorticoid injection, although comparable with new drugs in terms of pain relief [122], lacks evidence of delaying time to surgery [123].

Finally, collaterally to OA, a recent article investigated the feasibility and outcomes of ultrasound-guided percutaneous irrigation for painful calcific tendinopathy (US-PICT) outside the rotator cuff (including the knee) with optimal results in terms of safety and pain relief [124]. Noteworthy, patients affected by calcific tendinopathy can benefit from this treatment for pain relief and a reduction/suspension of anti-inflammatory and/or analgesic drugs [125,126,127].

### 2.6. Ultrasound and Artificial Intelligence (AI)

Radiomics has been frequently and successfully coupled with AI, and in particular with deep learning (DL) approaches in the musculoskeletal field.

Although clinical research first focused on MRI and CT applications of AI, the advance in US technology with ultra-high-resolution probes is slowly shifting the focus of researchers on this modality. In knee OA, US analysis of peri-articular soft-tissue of the knee was the main application.

For instance, DL-based US is employed to diagnose muscle diseases and segment muscle imaging. A CNN-based method proposed by Burlina et al. [128] was used to assess and classify inflammatory muscle diseases, improving the diagnostic accuracy of neuromuscular diseases. The accuracy of this CNN-based method compared to conventional ML methods for classifying three conditions of myositis was 76.2 ± 3.1% vs. 72.3 ± 3.3% (normal vs. affected), 86.6 ± 2.4% vs. 84.3 ± 2.3% (normal vs. inclusion body myositis), and 74.8 ± 3.9% vs. 68.9 ± 2.5% (inclusion body myositis vs. dermatomyositis or polymyositis). Chen et al. developed a CNN method for the automatic segmentation of the rectus femoris muscle, requiring only 0.2 s. Real-time US images of the rectus femoris muscle were obtained during muscle contraction, followed by feature extraction and fractional map reconstruction to build a CNN segmentation [129].

Potential future applications around the knee might include the detection and classification of extensor mechanism injury, assessment of tendon healing, or quantitative analysis of knee joint effusions or synovitis.

## 3. Conclusions

Conventional radiography is used in conjunction with a clinical examination to diagnose osteoarthritis of the knee, while MRI is used for additional diagnoses and as a second-level imaging tool. Over the past 20 years, there has been a steady increase in the scientific literature regarding the role of ultrasound imaging in the diagnosis of osteoarthritis. Ultrasound imaging is inexpensive, generally accessible, and frequently used in primary healthcare.

Ultrasound imaging has been suggested as a potential method for assessing changes in the knee joint. Additionally, the ultrasound has excellent repeatability and a strong correlation with MRI findings. Significantly, in some areas, including the detection of osteophytes, joint inflammation, meniscus protrusion, and localized cartilage degradation, ultrasound appears to perform even better than conventional radiography. In the clinical setting for osteoarthritis diagnosis, ultrasound can be genuinely viewed as an adjunctive technique to conventional radiography, according to the existing literature.

In the future, new technological advancements might even improve the ultrasound’s diagnostic value. Particularly, tele-ultrasonography may provide a simple instrument to provide accurate and repeated follow-up controls at this particular goal, alone or in association with other clinical tools [130]. Furthermore, raw radiofrequency data from ultrasound backscattering signals give chances for quantitative, in-depth assessments of cartilage and other articular structures. Future developments in applications based on ultrasound tools could improve their clinical and scientific utility. These tools are continually evolving, and so must the clinicians’ knowledge of them through constant research and the study of comprehensive narrative reviews. 

## Figures and Tables

**Table 1:** Studies comparing imaging-guided to blind injections in knee OA (CR: conventional radiography; HA: Hyaluronic acid; N: Number of participants; OA: Osteoarthritis; RCT: Randomised controlled trial; US: Ultrasonography).

Study	N. of Patients	Site	Study Design	Imaging	Outcome
Bum Park, 2012 [111]	99	Knee	RCT	US	Accuracy of HA injection vs. blind injection OR (95% CI) for an accurate injection with US compared with blind: 4.68 (0.94 to 23.30).
Im et al., 2009 [112]	99	Knee	RCT	US	Accuracy of HA injection vs. blind injection Accurate injections: 95.5% (US-guided) vs. 77.2% (blind); *p* = 0.01.
Jang et al., 2013 [113]	126	Knee	RCT	US	Accuracy of US-guided in plain injection, US-guided out-of-plane injections, and blind injection of triamcinolone hexacetonide Accuracy: US-guided in plain 95.1%; US-guided out-of-plane 97.7%; blind 78% *p* < 0.05 blind vs. US-guided injections
Sibbitt et al., 2011 [114]	92	Knee	RCT	US	US-guided vs. blind triamcinolone in terms of pain relief, pain related to the injection, reinjection rate, and cost Significant decrease in pain only in patients treated with US-guided injection; US-guided procedure was related to lower pain and reinjection rate, but higher costs.

## Data Availability

No new data were created or analyzed in this study. Data sharing is not applicable to this article.
